# 6-Gingerol Improves In Vitro Porcine Embryo Development by Reducing Oxidative Stress

**DOI:** 10.3390/ani13081315

**Published:** 2023-04-11

**Authors:** Wenjie Yu, Yanxia Peng, Xinyue Peng, Ze Li, Chang Liu, Liu Yang, Yan Gao, Shuang Liang, Bao Yuan, Chengzhen Chen, Nam-hyung Kim, Hao Jiang, Jiabao Zhang

**Affiliations:** 1 Department of Laboratory Animals, Jilin Provincial Key Laboratory of Animal Model, Jilin University, Changchun 130062, China; 2 School of Grains, Jilin Business and Technology College, Changchun 130507, China; 3 Tongyu Grassland Management Station, Changchun 137200, China; 4 Department of Animal Science, Chungbuk National University, Cheongju 361-763, Chungbuk, Republic of Korea

**Keywords:** 6-gingerol, porcine embryo, reactive oxidative stress, proliferation, apoptosis, autophagy

## Abstract

**Simple Summary:**

Excellent quality of early embryonic development contributes to a successful pregnancy. At present, most in vitro cultured embryos can only develop to the blastocyst stage at most, because an in vitro culture (IVC) system cannot replace the physiological environment in vivo. During IVC, excessive accumulated reactive oxygen species in embryos cannot be easily metabolized, which will cause oxidative stress and suppress embryo development. In this study, we found that anti-oxidation capacity of early embryo was improved by adding 6-gingerol to IVC. Moreover, 6-gingerol can also improve blastocyst rate, cell proliferation, mitochondrial function, inhibit cell apoptosis, autophagy, and regulate functional genes expression in blastocyst. These results are helpful to optimize the early embryo culture system, and thus provide a theoretical basis for improving the early embryo quality and the efficiency of subsequent pregnancy.

**Abstract:**

6-Gingerol, the main active ingredient in ginger, exhibits a variety of biological activities, such as antioxidant, anti-inflammatory, and anticancer activities, and can affect cell development. However, the effects of 6-gingerol on mammalian reproductive processes, especially early embryonic development, are unclear. This study explored whether 6-gingerol can be used to improve the quality of in vitro-cultured porcine embryos. The results showed that 5 μM 6-gingerol significantly increased the blastocyst formation rates of porcine early embryos. 6-Gingerol attenuated intracellular reactive oxygen species accumulation and autophagy, increased intracellular glutathione levels, and increased mitochondrial activity. In addition, 6-gingerol upregulated *NANOG*, SRY-box transcription factor 2, cytochrome c oxidase subunit II, mechanistic target of rapamycin kinase, and RPTOR independent companion of MTOR complex 2 while downregulating Caspase 3, baculoviral IAP repeat containing 5, autophagy related 12, and Beclin 1. Most importantly, 6-gingerol significantly increased the levels of p-extracellular regulated protein kinase 1/2 while reducing the levels of p-c-Jun N-terminal kinase 1/2/3 and p-p38. These results indicate that 6-gingerol can promote the development of porcine early embryos in vitro.

## 1. Introduction

In vitro embryo culture systems, including procedures for in vitro maturation (IVM), in vitro fertilization (IVF) and in vitro embryo culture (IVC), are currently widely used for embryonic developmental mechanism research, in vitro embryo production, and human assisted reproduction [1]. However, it is difficult to simulate the optimal embryonic growth environment in vitro, and embryo quality and subsequent development ability are still low, especially in porcine in vitro embryo production systems [2]. Therefore, continuous development and optimization of these culture systems are important for improving porcine early embryo quality [3].

The addition of hormones [4,5] and growth factors [6] can improve the quality of early embryo development in vitro. However, the overall efficiency of embryo production in vitro is still low [1,7]. One of the most important potential reasons is that reactive oxygen species (ROS) can inhibit the development of porcine embryos [8]. The oxygen concentration in the normal reproductive tract is lower than the oxygen concentration in air, and oxygen levels higher than those in the body can cause ROS accumulation [9]. Oxidative stress caused by ROS accumulation can easily cause DNA damage in cells and oxidative modification of proteins in cells, which further cause mitochondrial destruction and lead to cell death [10]. Therefore, it is critical to use antioxidants to inhibit oxidation during porcine embryonic development. Previous studies showed that antioxidants, such as Vitamin C and coenzyme Q10, are effective in reducing ROS accumulation during both in vivo and in vitro embryo development [11,12]. Exploring additional antioxidants and clarifying their mechanisms is meaningful for improving embryo quality.

Ginger is commonly used in functional dietary supplements, beverages, and foods (sugar products, jams, and pickled products) [13]. In Asia and some non-Asian countries, ginger has been used as a medicine to treat diseases such as arthritis, indigestion, diabetes, and irregular menstruation [14]. 6-Gingerol (6-G) extracted from ginger has the greatest abundance of biologically active compounds of any ginger component and exerts various pharmacological effects, including antioxidant, apoptosis- and autophagy-regulating, cell proliferation-promoting, and mitochondrial function-maintaining effects [15]. Importantly, 6-G exerts pharmacological effects by regulating the mitogen-activated protein kinase (MAPK) signaling pathway [16] and inhibits apoptosis to attenuate myocardial ischemia/reperfusion injury by activating the phosphatidylinositol 3-kinase/Akt and high mobility group box 2-c-Jun N-terminal kinase 1/2/3 (JNK1/2/3)-nuclear factor kappa B pathways [17]. However, few studies on the effects and mechanisms of 6-G on animal reproduction have been carried out, especially with regard to early embryos. Whether 6-G can be used as an antioxidant in in vitro embryo production and the possible related mechanisms are not clear.

Porcine parthenogenetically activated (PA) embryos can be acquired in a relatively short amount of time, they are also frequently used in early embryo development-related studies. Studies indicated that parthenogenetic embryos could retain the female’s genetic characteristics, which were used in studies of mitochondrial functions, production of autologous stem cells, and cytoplasmic activity [18,19]. In this study, the potential effects of 6-G on oxidative stress, cell proliferation, apoptosis, autophagy, mitochondrial function, and embryo quality were explored in PA embryos. These results are helpful to optimize the embryo IVC system, and thus provide a theoretical basis for improving the early embryo quality.

## 2. Materials and Methods

All chemicals and reagents were purchased from Sigma-Aldrich/Merck unless expressly stated otherwise. According to the Institutional Animal Care and Use Committee of Jilin University (IACUC-ID-201802070), all experiments were carried out in the Experimental Animal Center of the Jilin University.

### 2.1. Oocyte Collection and IVM

Cumulus-oocyte complexes were collected from 3–6 mm follicles of prepubertal gilt ovaries and placed in a 4-well culture plate with 500 μL of IVM medium for 44 h. The details are indicated in the Supplementary Materials.

### 2.2. Parthenogenetic Activation and In Vitro Embryo Culture

Mature oocytes were parthenogenetically activated. Next, the oocytes were cultured in IVC medium with cytochalasin B (#C6762) for 3 h. Then, the oocytes were transferred into IVC medium with/without 0 μM, 5 μM, 10 μM, and 20 μM 6-G (#S3836, Selleck Chemicals, Shanghai, China). The rate of blastocyst formation was calculated as the ratio of the number of blastocysts to the number of cleavages. The details are indicated in the Supplementary Materials.

### 2.3. Cell Proliferation Analysis

Briefly, embryonic cell proliferation capacity was analyzed by a 5-ethynyl-2′-deoxyuridine (EdU) assay with a BeyoClick™ EdU Cell Proliferation Kit (#C0075; Beyotime, Shanghai, China). The cell proliferation rate was calculated as the number of EdU-positive cells to the total number of cells in blastocysts. The details are indicated in the Supplementary Materials.

### 2.4. Terminal Deoxynucleotidyl Transferase-Mediated dUTP-Biotin Nick End-Labeling (TUNEL) Assays

Briefly, the cell apoptosis level was analyzed by an In Situ Cell Death Detection Kit (#11684795910; Roche, Mannheim, Germany). The ratio of the number of TUNEL-positive nuclei to the total number of nuclei was calculated as the apoptosis rate. The details are indicated in the Supplementary Materials.

### 2.5. ROS and Glutathione (GSH) Assays

Briefly, 4-cell-stage porcine embryos were incubated in 10 μM 2′,7′-dichlorodihydrofluorescein diacetate (DCFH; #C2938; Invitrogen, Rochester, NY, USA) and 10 μM 4-chloromethyl-6,8-difluoro-7-hydroxycoumarin (CMF_2_HC; #C12881; Invitrogen), respectively. The details are indicated in the Supplementary Materials.

### 2.6. Mitochondrial Membrane Potential (MMP, ΔΨm) Assay

Briefly, 4-cell-stage embryos were incubated in 2 μM 5,5′,6,6′-tetrachloro-1,1′,3,3′-tetraethylbenzimidazolylcarbocyanine-iodide dye (JC-1; #M34152; Invitrogen). The details are indicated in the Supplementary Materials.

### 2.7. Determination of ATP Levels

Briefly, the ATP levels in 4-cell-stage embryos were measured using an ATP Determination Kit (#A22066; Invitrogen). The details are indicated in the Supplementary Materials.

### 2.8. Immunofluorescence

Briefly, embryos were fixed in 4% paraformaldehyde and permeabilized with 0.3% Triton X-100. Then, the embryos were blocked in 5% BSA and incubated with a primary antibody against microtubule-associated protein 1 light chain 3 beta (LC3B; #ab63817; Abcam, Cambridge, MA, USA). Then, the embryos were incubated with a secondary antibody (#ab150073; Abcam) for 1 h. The nuclei were stained with Hoechst 33342. The details are indicated in the Supplementary Materials.

### 2.9. RNA Extraction and Quantitative Real-Time Reverse Transcription Polymerase Chain Reaction (qRT-PCR) Analysis

Briefly, mRNA was extracted using the Dynabeads mRNA DIRECT Purification Kit (#61011; Invitrogen). A TIANScript First Strand cDNA Synthesis Kit (#KR118; Tiangen Biotech Co., Beijing, China) was used to synthesize cDNA. Gene expression was quantified using the 2^−ΔΔCt^ method with *18S rRNA* as the standard. The details are indicated in the Supplementary Materials and all the primers are listed in Supplementary Table S1.

### 2.10. Western Blot Analysis

Briefly, a RIPA lysis buffer (#R0010; Solarbio, Beijing, China) was used for total protein extraction. Proteins were separated by SDS-PAGE and transferred to polyvinylidene fluoride membranes (0.45 μm; #IPVH00010; Millipore, Bedford, MA, USA). The membranes were blocked with 5% BSA and incubated with a primary antibody. Then, the membranes were incubated with secondary antibodies. The details are indicated in the Supplementary Materials and the antibody information is shown in Supplementary Table S2.

### 2.11. Statistical Analysis

All calculations were performed using SPSS software v.22.0 (SPSS, Inc., Chicago, IL, USA). Data from two groups were compared using the Student’s *t*-test. Tests with three or more means were analyzed using a one-way ANOVA (Tukey-Kramer). All data are presented as mean ± SD. The total numbers of embryos used (*N*) in each group and replicates (*R*) in each experiment are shown in the results and figure legends. *p* < 0.05 and *p* < 0.01 were considered to indicate significant differences.

## 3. Results

### 3.1. 6-G Improved the Blastocyst Formation Rate

**Figure 1:**
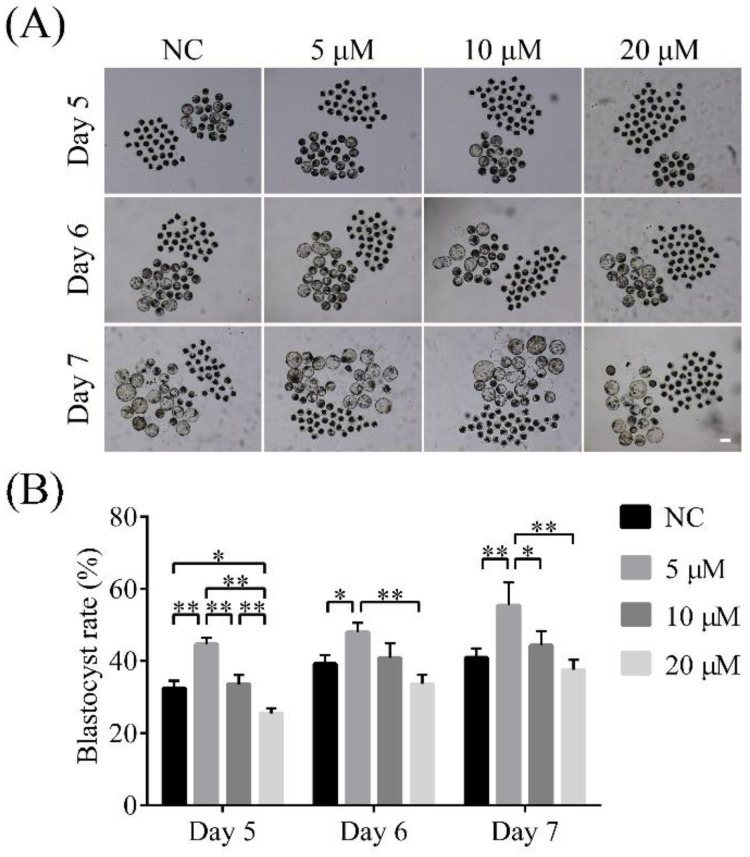
Effects of 6-gingerol (6-G) on blastocyst formation rate of porcine early embryos. (**A**) Representative images of embryos on day 5, day 6, and day 7 treated with 0 μM, 5 μM, 10 μM, or 20 μM 6-G, respectively. Scale bar = 200 μm. (**B**) Blastocyst formation rates on day 5, day 6, and day 7 treated with 0 μM, 5 μM, 10 μM, or 20 μM 6-G, respectively. *R* = 3. * *p* < 0.05; ** *p* < 0.01.

To explore the effects of 6-G on early embryo development, we incubated parthenogenetic porcine early embryos with different 6-G concentrations (0, 5, 10, and 20 μM). As shown in Figure 1, the blastocyst formation rates were higher in the 5 μM 6-G-supplied group than in the other groups at day 5, day 6, and day 7. On day 5, the blastocyst formation rates were 32.46% ± 2.06% (*N* = 173), 44.67% ± 1.77% (*N* = 177, *p* < 0.01), 33.56% ± 2.54% (*N* = 176, *p* = 0.903), and 25.45% ± 1.42% (*N* = 181, *p* = 0.011) with 0 μM, 5 μM, 10 μM, and 20 μM 6-G, respectively. On day 6, these rates were 39.23% ± 2.42% (*N* = 173), 48.04% ± 2.54% (*N* = 177, *p* = 0.027), 40.88% ± 3.99% (*N* = 176, *p* = 0.899), and 33.62% ± 2.57% (*N* = 181, *p* = 0.171) with the aforementioned 6-G concentrations, respectively, and on day 7, these rates were 40.90% ± 2.55% (*N* = 173), 54.78% ± 5.46% (*N* = 177, *p* < 0.01), 44.30% ± 3.96% (*N* = 176, *p* = 0.711), and 37.57% ± 2.77% (*N* = 181, *p* = 0.724), respectively. Therefore, a concentration of 5 μM was selected for further research.

### 3.2. 6-G Enhanced Cell Proliferation

**Figure 2:**
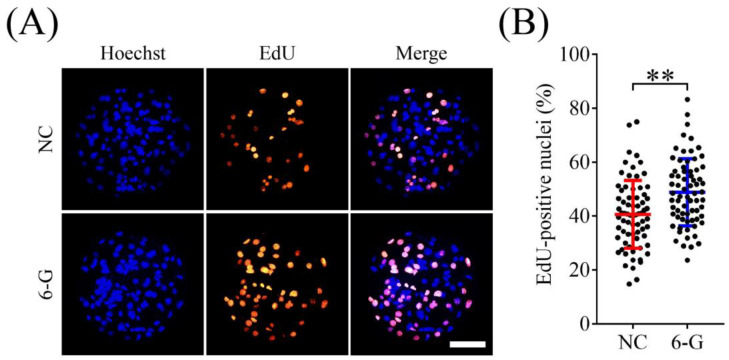
Effects of 6-gingerol (6-G) on cell proliferation in blastocysts. (**A**) Representative 5-ethynyl-2′-deoxyuridine (EdU) staining images of embryos with or without 6-G at day 7. (**B**) Proportions of proliferating cells to total numbers of cells in embryos with or without 6-G treatment. *R* = 5. Bar = 100 μm. ** *p* < 0.01.

Subsequently, we examined the effect of 6-G on proliferation in early embryos by the EdU assay. As shown in Figure 2, compared with the NC group, the 6-G treatment group exhibited a significantly higher proportion of proliferating cells among the total number of cells (48.87% ± 12.46% (*N* = 70) versus 40.66% ± 12.57% (*N* = 71), *p* < 0.01). Based on the above results, 6-G can enhance cell proliferation during early embryo development.

### 3.3. 6-G Reduced Apoptosis of Porcine Embryos

**Figure 3:**
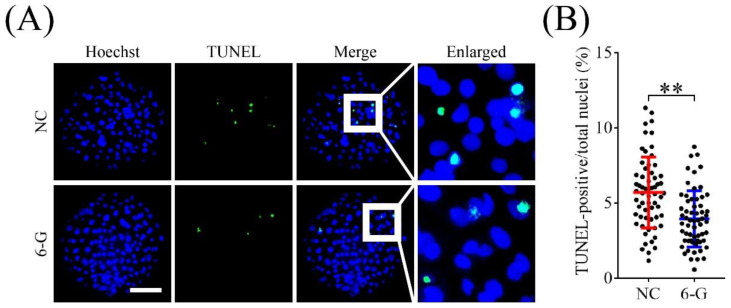
Effects of 6-gingerol (6-G) on cell apoptosis in blastocysts. (**A**) Representative staining images of Hoechst and terminal deoxynucleotidyl transferase-mediated dUTP-biotin nick end-labeling (TUNEL) of blastocysts. Bar = 100 μm. (**B**) Apoptotic rates in blastocysts with or without 6-G treatment. *R* = 5. ** *p* < 0.01.

As shown in Figure 3, the proportion of TUNEL-positive nuclei in the 6-G-treated group was significantly lower than that in the NC group (3.94 ± 1.87% (*N* = 61) versus 5.70 ± 2.36% (*N* = 62), *p* < 0.01). This suggests that treatment with 6-G reduces apoptosis in blastomeres of porcine embryos.

### 3.4. 6-G Enhanced Antioxidant Capacity in Porcine Embryos

**Figure 4:**
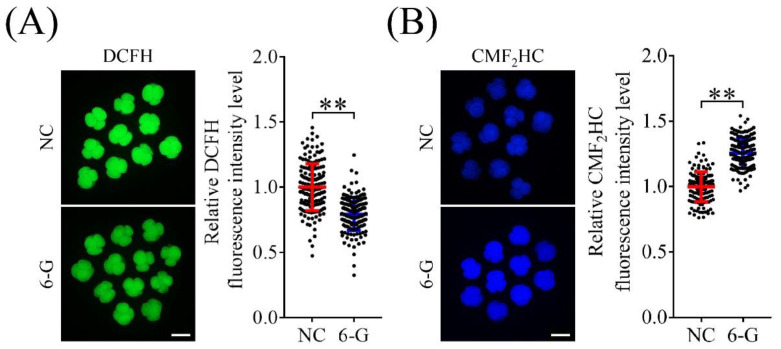
Effects of 6-gingerol (6-G) on reactive oxygen species (ROS) and glutathione (GSH) levels. (**A**) Representative 2′,7′-dichlorodihydrofluorescein diacetate (DCFH) staining images of 4-cell-stage embryos with or without 6-G. The relative DCFH fluorescence intensity decreased significantly in 4-cell-stage embryos with 6-G. *R* = 5. Bar = 100 μm. (**B**) Representative 4-chloromethyl-6,8-difluoro-7-hydroxycoumarin (CMF_2_HC) staining images of 4-cell-stage embryos with or without 6-G. The relative CMF_2_HC fluorescence intensity increased significantly in 4-cell-stage embryos with 6-G. *R* = 5. Bar = 100 μm. ** *p* < 0.01.

To investigate whether 6-G can inhibit ROS accumulation, we used DCFH fluorescent probes to detect ROS levels in 4-cell-stage embryos. As shown in Figure 4, the fluorescence intensities of DCFH in blastomeres were significantly lower (0.79 ± 0.13-fold; *N*_NC_ = 163, *N*_6-G_ = 168; *p* < 0.01) in the 6-G treatment group than in the NC group (Figure 4A). In addition, we analyzed the relative CMF_2_HC level to detect GSH levels in 4-cell-stage embryos and found that they were significantly higher (1.26 ± 0.12-fold; *N*_NC_ = 140, *N*_6-G_ = 155; *p* < 0.01) in the 6-G treatment group than in the NC group (Figure 4B).

### 3.5. 6-G Improved Mitochondrial Function

**Figure 5:**
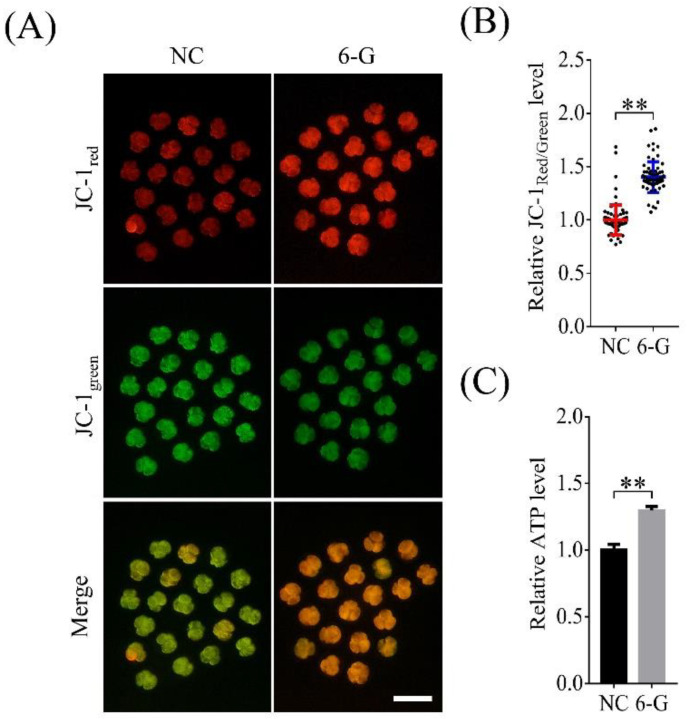
Effects of 6-gingerol (6-G) on mitochondrial function in 4-cell-stage embryos. (**A**) Representative images of 5,5′,6,6′-tetrachloro-1,1′,3,3′-tetraethylbenzimidazolylcarbocyanine-iodide dye (JC-1) in the NC and 6-G treatment groups. Bar = 200 μm. (**B**) Relative JC-1_Red/Green_ fluorescence intensity ratios with or without 6-G treatment. *R* = 4. (**C**) Relative ATP level change in 4-cell-stage embryos after 6-G treatment. *R* = 3. ** *p* < 0.01.

Mitochondrial activity directly affects embryonic cell proliferation and developmental potential. To study whether 6-G can improve mitochondrial function in porcine early embryos, we measured MMP in 4-cell-stage embryos. As shown in Figure 5A, the JC-1_Red/Green_ fluorescence intensity ratio in 4-cell-stage embryos was significantly higher (1.40 ± 0.14-fold; *N*_NC_ = 81, *N*_6-G_ = 70; *p* < 0.01) in the 6-G-treated group than in the NC group (Figure 5B). In addition, the ATP level in 6-G treated embryos was also higher (1.30 ± 0.03-fold; *N*_NC_, *N*_6-G_ = 270; *p* < 0.01) than that in non-6-G-treated embryos (Figure 5C).

### 3.6. 6-G Inhibited Autophagy in Blastocysts

**Figure 6:**
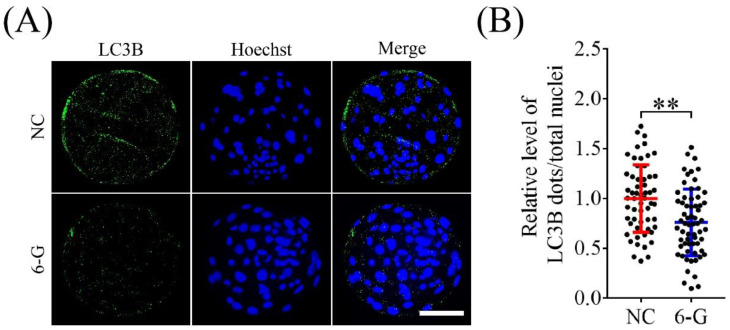
6-Gingerol (6-G) treatment affects the autophagy levels in porcine early embryos. (**A**) Representative immunofluorescence images of microtubule-associated protein 1 light chain 3 beta (LC3B) in blastocysts. Bar = 100 μm. (**B**) Proportions of LC3B-positive puncta in blastocysts with or without 6-G treatment. *R* = 5. ** *p* < 0.01.

Excessive ROS production can induce autophagy in cells. Therefore, we analyzed the autophagy levels of embryos after 6-G treatment. As shown in Figure 6A, compared with the NC group, the 6-G group exhibited a significantly lower number of intracellular LC3B-positive puncta (0.76 ± 0.33-fold; *N*_NC_ = 57, *N*_6-G_ = 64; *p* < 0.01). This finding indicates that 6-G may reduce intracellular autophagy levels.

### 3.7. 6-G Regulated Embryo Pluripotency, Apoptosis, Autophagy, and Proliferation-Related Gene Expression in Blastocysts

**Figure 7:**
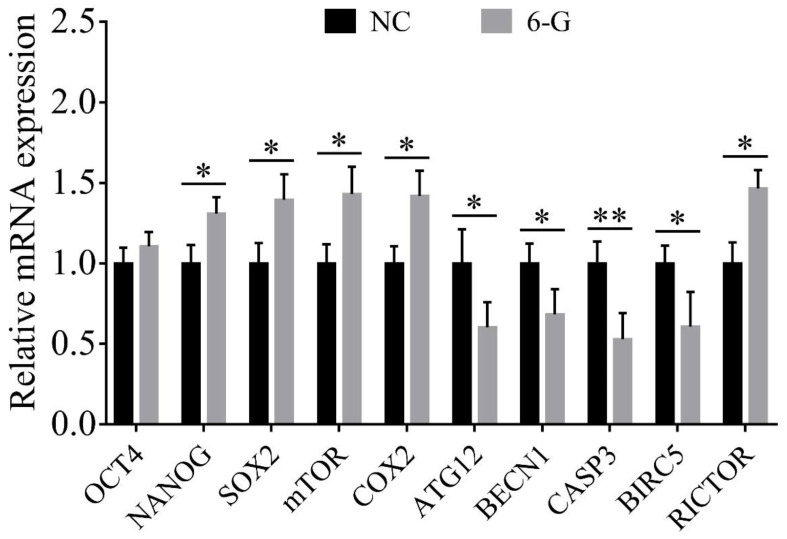
Results of quantitative real-time reverse transcription polymerase chain reaction (qRT-PCR) analysis of functional gene expression. Expression of embryo pluripotency- (*OCT4*; *NANOG*; and SRY-box transcription factor 2, *SOX2*), proliferation- (mechanistic target of rapamycin kinase, *mTOR*), hatching (cytochrome c oxidase subunit II, *COX2*), apoptosis- (RPTOR independent companion of MTOR complex 2, *RICTOR*; caspase 3, *CASP3*; and baculoviral IAP repeat containing 5, *BIRC5*), and autophagy- (autophagy related 12, *ATG12*; beclin 1, *BECN1*) related genes were all detected in blastocysts at day 7. *R* = 3. * *p* < 0.05; ** *p* < 0.01.

To study the potential mechanisms by which 6-G promotes early embryo development, we examined the expression of related genes (shown in Figure 7). Compared with NC embryos, 6-G-treated embryos exhibited significant upregulation of embryo pluripotency- (*NANOG*, 1.31 ± 0.10-fold, *p* = 0.039; SRY-box transcription factor 2, *SOX2*, 1.39 ± 0.16-fold, *p* = 0.047; *N*_NC_, *N*_6-G_ = 90), proliferation- (mechanistic target of rapamycin kinase, *mTOR*, 1.43 ± 0.17-fold, *p* = 0.039; *N*_NC_, *N*_6-G_ = 90), hatching- (cytochrome c oxidase subunit II, *COX2*, 1.42 ± 0.16-fold, *p* = 0.032; *N*_NC_, *N*_6-G_ = 90) related genes, and the apoptosis-related gene RPTOR independent companion of MTOR complex 2 (*RICTOR*, 1.46 ± 0.11-fold, *p* = 0.018; *N*_NC_, *N*_6-G_ = 90) and significant downregulation of the apoptosis- (caspase 3, *CASP3*, 0.53 ± 0.16-fold, *p* < 0.01; baculoviral IAP repeat containing 5, *BIRC5*, 0.61 ± 0.22-fold, *p* = 0.023; *N*_NC_, *N*_6-G_ = 90) and the autophagy- (autophagy related 12, *ATG12*, 0.60 ± 0.16-fold, *p* = 0.028; beclin 1, *BECN1*, 0.68 ± 0.16-fold, *p* = 0.030; *N*_NC_, *N*_6-G_ = 90) related genes. However, 6-G did not significantly increase the expression level of *OCT4* (1.10 ± 0.09-fold, *p* = 0.256; *N*_NC_, *N*_6-G_ = 90).

### 3.8. 6-G Regulated MAPKs Activations in Blastocysts

**Figure 8:**
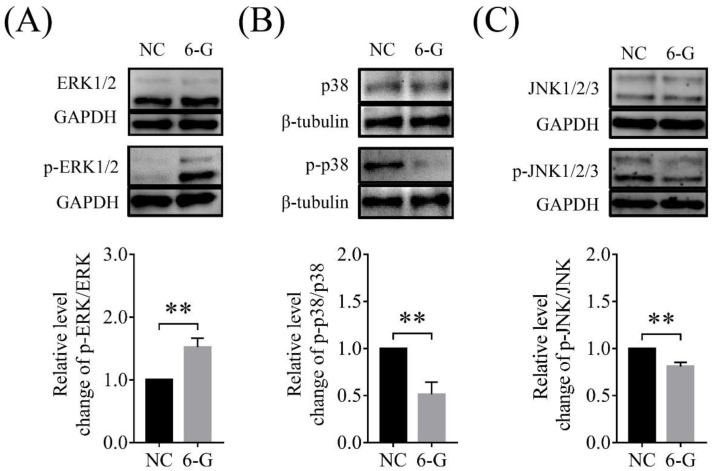
Effects of 6-gingerol (6-G) on the extracellular regulated protein kinase 1/2 (ERK1/2), c-Jun N-terminal kinase 1/2/3 (JNK1/2/3), and p38 pathways. (**A**) The level of p-ERK1/2 in the 6-G treatment group was higher than that of the NC group. *R* = 3. (**B**) The level of p-p38 in the 6-G treatment group was lower than that of the NC group. *R* = 3. (**C**) The level of p-JNK1/2/3 in the 6-G treatment group was lower than that of the NC group. *R* = 3. ** *p* < 0.01.

The MAPK signaling pathway can regulate cell proliferation, differentiation, apoptosis, and autophagy. Therefore, we used Western blotting to detect the effects of 6-G treatment on the extracellular regulated protein kinase 1/2 (ERK1/2), JNK1/2/3, and p38 signaling pathways. As shown in Figure 8, the addition of 6-G significantly increased the level of p-ERK1/2 (1.52 ± 0.14-fold, *p* < 0.01; *N*_NC_, *N*_6-G_ = 90; Figure 8A), while reducing the levels of p-p38 (0.51 ± 0.13-fold, *p* < 0.01; *N*_NC_, *N*_6-G_ = 90; Figure 8B), and p-JNK1/2/3 (0.81 ± 0.05-fold, *p* < 0.01; *N*_NC_, *N*_6-G_ = 90; Figure 8C).

## 4. Discussion

Studies have shown that 6-G alleviates oxidative damage and inflammation in the ovaries [20,21]. Porcine early embryos are more sensitive to oxidative stress during in vitro embryo production than in vivo due to their unique structures [22]. The results of this study indicate that the addition of 6-G can effectively improve the developmental potential of early porcine embryos.

In this study, 6-G treatment effectively reduced the accumulation of ROS in blastomeres at the 4-cell stage, likely because 6-G can effectively prevent -OH-induced DNA damage, in particular by scavenging various free radicals [23]. This effect was likely mediated by 6-G-induced high levels of GSH. Intracellular ROS levels are highly correlated with GSH levels. The findings show that when ROS levels in the body are increased, 6-G-mediated enhancement of the GSH defense system can increase the removal of excess oxygen free radicals and maintain dynamic redox balance. These results are consistent with previous suggestions that 6-G can alleviate ROS-induced diseases and conditions by increasing GSH levels [24]; such conditions include impaired ovarian follicle development and abnormal fertilization [25].

At the 4-cell stage, zygotic genome activation is essential for porcine early embryo development and pregnancy [26,27]. During IVC of embryos, activation of the zygotic genome generally occurs 24–48 h after parthenogenetic activation or IVF. This stage is more sensitive than other stages to negative conditions in the external environment, especially the accumulation of ROS, which leads to the failure of zygotic genome activation and leads to the suppression of embryo development [28]. The results indicate that 6-G may exert biological effects at least as early as at this stage.

Mitochondria play essential roles during embryo development both before and after zygotic genome activation [29]. Excessive accumulation of ROS causes oxidative damage, MMP depolarization [30], aging, and apoptosis [31]. In this study, we found that 6-G supplementation significantly increased the MMP and ATP level in blastomeres at the 4-cell stage, improved the ability of embryos to proliferate and effectively reduced the number of apoptotic cells in the embryos. This result is consistent with previous findings that 6-G inhibits disruption of MMP [32] and is probably related to the roles of 6-G in increasing the activity of the mitochondrial enzymes NADH oxidase, succinate dehydrogenase, and Sirtuin 3 [33]. Previous studies have also shown that 6-G can reduce intracellular ROS levels by regulating nuclear factor kappa B translocation [34]. In addition, 6-G upregulated antioxidant enzymes, such as glyoxylate carboligase and heme oxygenase 1, and further protects against cytotoxicity and apoptotic cell death resulting from processes such as ROS-induced DNA fragmentation, disruption of MMP, and autophagy. These effects seem to be mediated by regulation of nuclear factor 2 [35], p38 MAPK, JNK [36], and phosphatidylinositol 3-kinase/Akt [37]. In this study, we found that 6-G increased p-ERK levels and decreased p-JNK and p-p38 levels. This indicates that 6-G exerts a regulatory effect on cell survival or apoptosis by affecting the dynamic balance between the growth factor-activated ERK pathway and the stress-activated JNK-p38 pathway [38]. On the one hand, this regulation depends on ERK pathway-mediated direct targeting and regulation of the cell cycle and indirect regulation of RNA metabolism and transport [39]. On the other hand, it also depends on the regulation of the JNK pathway by various cellular stress and growth factors [40]. 6-G may enable the effects mediated by these pathways to regulate biological processes, such as cell morphology changes, immune responses, and apoptosis [41,42].

To explore the potential mechanism by which 6-G promotes embryo development, we also examined functional gene expression changes. The results showed that the addition of 6-G significantly upregulated the anti-apoptotic gene *RICTOR* and downregulated the pro-apoptotic gene *CASP3*. These factors help stabilize the intracellular environments of blastomeres. The results are consistent with the findings that 6-G can improve embryo development and reduce apoptosis. Early embryos with stable cell states and enhanced mitochondrial function show high proliferation ability and reduced nuclear apoptosis rates [43,44]. Notably, the quality of blastomeres is an important basis for embryo development before implantation [45]. Combined with the result that 6-G improved the proliferation-related gene *mTOR* [46], these findings suggest that 6-G can stabilize or even optimize the intracellular environment of blastomeres, creating a relatively stable internal environment and promoting cell proliferation and development. This study also revealed that the addition of 6-G can significantly increase the cell proliferation capacity in blastocysts. Interestingly, 6-G significantly decreased the expression level of the apoptosis-related gene *BIRC5*. *BIRC5* is widely known as an anti-apoptosis gene. However, a previous study has found that overexpression of *BIRC5* significantly inhibits cell survival [47]. In addition, compared with those in unfrozen bovine blastocysts, the levels of apoptosis and the expression of apoptosis-related genes *BIRC5* and *CASP3* were also significantly increased in frozen embryos [48], which was similar to our results that 6-G reduced the level of apoptosis and downregulated the expression levels of *BIRC5* and *CASP3* in porcine blastocysts. We speculate that this may be because the addition of 6-G improves the embryonic development environment while the high level of anti-apoptotic factors is not needed. In addition, embryo pluripotency-related genes (*NANOG* and *SOX2*) were significantly upregulated in this study, which indicates that 6-G has the potential to promote establishment of the epiblast and hypoblast [49], improve the quality of inner cell masses [50,51], and stabilize the basic functions of embryonic stem cells [52,53]. However, there are some reports showing that there are differences in the expression of *NANOG* and *SOX2* during embryo development not only between embryos produced in vivo and in vitro, but also between parthenogenetic and IVF embryos [54]. Additional research about the roles of 6-G in IVF and somatic cell nuclear transfer embryos is still needed. The upregulation of *COX2* observed in this study suggests that 6-G has potential effects on early embryo implantation and decidualization [55,56]. Our results showed that the expression levels of autophagy-related genes *ATG12* and *BECN1* with 6-G treatment. ATG12 can target the elongation of the autophagosome membrane. A previous study showed that inhibition of *ATG12* significantly decreased the autophagy level [57]. *BECN1* also plays important roles in autophagy [58]. However, mTOR plays opposite roles in the regulation of autophagy [59]. The early stages of the autophagic process are inhibited by mTOR, and mTOR can also regulate the lysosomal degradative capacity by preventing the transactivation of genes encoding catalytic, regulatory, and structural factors [60]. These results were related to the inhibition of autophagosome formation with 6-G treatment, indicating that 6-G can significantly inhibit the autophagy level in porcine early embryos. Moreover, autophagy is one of the most important factors in mediating apoptosis [61], which indicates that the decreased level of autophagy with 6-G treatment may also be one of the reasons for the inhibition of apoptosis.

Conversely, some studies have shown that 6-G can play roles in inducing ROS production, reducing mitochondrial function, and promoting autophagy and apoptosis. However, such studies have mostly focused on cancer research [62]. In addition, we believe that different effects are caused by different concentrations of 6-G. 6-G (20 µM) inhibited blastocyst formation. This is similar to the results showing that an appropriate concentration of antioxidant supplements could improve early embryo development by reducing oxidative stress, while they would inhibit early embryo development at a high concentration [63,64,65]. This may be because oxidative stress signaling is also required during early embryonic development. A large amount of 6-G will reduce the ROS level too much. Reductive stress is just as dangerous as oxidative stress [66]. At the same time, the concentration of 6-G in the culture medium may decrease with time. However, during the 7 days of in vitro culture, the corresponding concentration of 6-G still affected the embryonic development. Furthermore, many studies have shown that biological substances can induce ROS production and promote apoptosis in cancer cells while exerting beneficial effects on normal cells, promoting cell proliferation and enhancing embryo development [67]. These different effects are related mainly to the abnormal physiological statuses of cancer cells, such as their abnormal gene expression (including that of insulin-like growth factor-1, DNA methyltransferase 1, and histone deacetylases) [68], enzyme activity [69], and activation of signaling pathways including the AKT, MAPK, and nuclear factor kappa B pathways [70]. In this study, 6-G was found to regulate the p38, JNK, and ERK pathways. However, how 6-G regulates key downstream genes through these pathways during early embryo development still needs further research.

In summary, our results indicate that the addition of 6-G to IVC systems increases GSH and regulates functional gene expression and the ERK, JNK, and p38 signaling pathways, thereby reducing intracellular ROS accumulation, autophagy, and apoptosis, and enhancing mitochondrial activity and cell proliferation to improve porcine preimplantation embryo development and competence. Our findings will suggest new methods and provide a theoretical basis for improving the quality of embryo development in vivo and in vitro.

## 5. Conclusions

6-G improved blastocyst rate, cell proliferation, mitochondrial function, and inhibited cell apoptosis and autophagy by reducing oxidative stress. Moreover, 6-G can regulate related gene expressions in blastocyst.

## Data Availability

The data presented in this study are available in the article or supplementary material.

## Supplementary Materials

The following supporting information can be downloaded at: https://www.mdpi.com/article/10.3390/ani13081315/s1, Table S1: Primer sequence for qRT-PCR; Table S2: Antibodies used for Western blotting; Supplementary Materials and Methods.
