# Supplementary material for: 6-Gingerol Improves In Vitro Porcine Embryo Development by Reducing Oxidative Stress
Source: Animals (Basel). 2023 Apr 11;13(8):1315. doi: 10.3390/ani13081315 (PMC10135256; doi:10.3390/ani13081315)
Supplement: Supplementary file 1 [file animals-13-01315-s001.zip › animals-2275341-supplementary.pdf]

# *Supplementary Materials*

Supplementary Tables ........................................................................................S-1

Table S1. Primer sequence for qRT-PCR ..........................................................S-1

Table S2. Antibodies used for Western blotting ................................................S-2

Supplementary Materials and Methods ....................................................................S-3

1. Oocyte Collection and IVM ...........................................................................S-3

2. Parthenogenetic Activation and *In Vitro* Embryo Culture .............................S-3

3. Cell Proliferation Analysis ..............................................................................S-3

4. TUNEL Assays ...............................................................................................S-4

5. ROS and GSH Assays .....................................................................................S-4

6. MMP Assay ....................................................................................................S-4

7. Determination of ATP Levels...........................................................................S-4

8. Immunofluorescence .......................................................................................S-4

9. qRT-PCR Analysis............................................................................................S-5

10. Western Blot Analysis ....................................................................................S-5

animals MDPI

# *Supplementary Tables*

**Supplementary Table S1. Primer sequence for qRT-PCR.**

| Genes | NCBI Accession No. | Sequences (5'to 3') | Product size (bp) |
|---|---|---|---|
| *18S rRNA* | NR_046261.1 | **F:** GCCCGAAGCGTTTACTTTGA<br>**R:** CCGCGGTCCTATTCCATTATT | 93 |
| *RICTOR* | XM_021076928.1 | **F:** GGTGCTAAAATTGAAAGTGG<br>**R:** TGCTTGTGTCCTCTCTACCT | 82 |
| *CASP3* | NM_214131.1 | **F:** GAGGCAGACTTCTTGTATGC<br>**R:** ACAAAGTGACTGGATGAACC | 93 |
| *BIRC5* | NM_214141.1 | **F:** CCTGGCAGCTCTACCTCAAG<br>**R:** GAAAGCACAACCGGATGAAT | 233 |
| *OCT4* | NM_001113060.1 | **F:** GTGAGAGGCAACCTGGAGAG<br>**R:** TCGTTGCGAATAGTCACTGC | 165 |
| *NANOG* | NM_001129971.1 | **F:** CATGAGTGTGGATCCAGCTTG<br>**R:** CCTGAATAAGCAGATCCATGG | 191 |
| *SOX2* | NM_001123197.1 | **F:** AAGAGAACCCCAAGATGCACAACT<br>**R:** GCTTGGCCTCGTCGATGAAC | 105 |
| *COX2* | NM_214321.1 | **F:** GGCTGCGGGAACATAATAGA<br>**R:** GCAGCTCTGGGTCAAACTTC | 183 |
| *mTOR* | XM_003127584.6 | **F:** AGGAGACCTCCTTTAACCAG<br>**R:** ATGTACTTCCTGCACCACTC | 60 |
| *BECN1* | NM_001044530.1 | **F:** TTTTCTGGGACAACAAGTTT<br>**R:** CAACCTCTTCTTTGAACTGC | 75 |
| *ATG12* | NM_001190282.1 | **F:** TGATGACAGTTATTTGGGCTAC<br>**R:** CAAAGTCCTCGCTGCTCT | 92 |

Annealing temperature of all reactions is 60 °C.
F: forward primer; R, reverse primer.

**Supplementary Table S2. Antibodies used for Western blotting.**

| Antibody | Company[a] | Cat. Number # |
|---|---|---|
| ERK | Abcam | 17942 |
| p-ERK | Cell Signaling Technology | 4370S |
| JNK | Abcam | 179461 |
| p-JNK | Abcam | 124956 |
| p38 | Abcam | 31828 |
| p-p38 | Cell Signaling Technology | 4511S |
| GAPDH | Cell Signaling Technology | 2118S |
| β-tubulin | ProteinTech | 10094-I-AP |
| Goat anti-Rabbit IgG (H+L)-HRP | Bioworld Technology | BS13278 |
| Goat anti-Mouse IgG (H+L)-HRP | Bioworld Technology | BS12478 |

[a] Abcam, Cambridge, MA, USA;
Cell Signaling Technology, Beverly, MA, USA;
Proteintech, Chicago, IL, USA;
Bioworld Technology Inc, Louis Park, MN, USA.

# Supplementary Materials and Methods

## 1. Oocyte Collection and IVM

Prepubertal gilt ovaries obtained from a local slaughterhouse were stored in saline at 30-35 °C and transferred to the laboratory within 1 h. Cumulus-oocyte complexes were aspirated from 3-6 mm follicles using a 10 mL syringe. After three washes in Tyrode's lactate HEPES, oocytes with more than three layers of cumulus cells were selected, placed in a 4-well culture plate with 500 µL of maturation medium (M199 (#M4530) with 10 ng/mL epidermal growth factor (#SRP3196), 1 µg/mL insulin (#I3536), 75 µg/mL kanamycin (#E004000), 0.91 mM sodium pyruvate (#P4562), 0.5 µg/mL follicle stimulating hormone (#110254629, Ningbo Second Hormone Factory, Ningbo, Zhejiang), 0.5 µg/mL luteinizing hormone (#110254634, Ningbo Second Hormone Factory), and 10% porcine follicular fluid), covered with mineral oil (#M8410), and cultured at 38.5 °C in an atmosphere of 5% $CO_2$ and 100% humidity for 44 h.

## 2. Parthenogenetic Activation and In Vitro Embryo Culture

Mature oocytes were denuded by using 0.1% hyaluronidase (#H3506) and parthenogenetically activated using two direct-current pulses of 120 V for 60 µs in 297 mM mannitol (#M9456) containing 0.1 mM $CaCl_2$ (#M7902), 0.05 mM $MgSO_4$ (#M2643), 0.1% polyvinyl alcohol (PVA; #P1763), and 0.5 mM HEPES (#H3784). Next, the oocytes were cultured in bicarbonate-buffered porcine zygote medium 5 containing 4 mg/mL BSA (#a8806) and 7.5 µg/mL cytochalasin B (#C6762) for 3 h to suppress the extrusion of the pseudo-second polar body. Then, the oocytes were transferred into a four-well plate; each well contained 500 µL of in vitro culture (IVC) medium with/without 0 µM, 5 µM, 10 µM, and 20 µM 6-G (#S3836, Selleck Chemicals, Shanghai, China) covered with mineral oil. The oocytes were incubated at 38.5 °C in an atmosphere of 5% $CO_2$ and 100% humidity without changing the medium. The rate of blastocyst formation was calculated as the ratio of the number of blastocysts to the number of cleavages.

## 3. Cell Proliferation Analysis

Embryonic cell proliferation capacity was analyzed by 5-ethynyl-2'- deoxyuridine (EdU) assay with a BeyoClick™ EdU Cell Proliferation Kit (#C0075; Beyotime, Shanghai, China). Briefly, embryos at the beginning of day 7 were transferred into pre-equilibrated IVC medium containing 10 µM EdU for 3 h. Next, the embryos were washed with PBS-PVA, fixed for 30 min in 4% paraformaldehyde in PBS-PVA and permeabilized with 0.3% Triton X-100 (#93443) in PBS-PVA for 30 min at room temperature. The embryos were then washed and incubated in a premade Click Reaction Solution (containing 860 µL of Click Reaction Buffer, 40 µL of $CuSO_4$, 2 µL of Azide-555, and 100 µL of Click Additive Solution) for 30 minutes at room temperature in the dark. The nuclei were labeled with 10 µg/mL Hoechst 33342 (#H3570; Thermo Fisher, Waltham, MA, USA) for 10 minutes at room temperature. After washing 3 times with PBS-PVA, the embryos were fixed on glass slides. Finally, a confocal laser scanning microscope (Carl Zeiss, Jena, Germany) was used to capture the fluorescence signals. ImageJ software (NIH, MD, USA) was used to analyze the numbers of EdU-positive cells and the total numbers of cells in blastocysts.

*4. TUNEL Assays*

Porcine embryos at day 7 were washed with PBS-PVA 3 times. The blastocysts were then fixed in 4% paraformaldehyde (#158127) for 30 minutes, washed 3 times with PBS-PVA, and treated with 0.3% Triton X-100 for 30 minutes. Then, the embryos were incubated with fluorescein-conjugated dUTP and the terminal deoxynucleotidyl transferase enzyme of an In Situ Cell Death Detection Kit (#11684795910; Roche, Mannheim, Germany) for 1 h in the dark at 37 °C. After washing twice with PBS-PVA, the cells were incubated with 10 μg/mL Hoechst 33342 for 15 minutes to label the nuclei. Finally, fluorescence images were taken using a fluorescence microscope and analyzed using ImageJ software (NIH). The ratio of the number of TUNEL-positive nuclei to the total number of nuclei was calculated as the apoptosis rate.

*5. ROS and GSH Assays*

Four-cell-stage porcine embryos at day 2 were washed 3 times and incubated in PBS-PVA containing 10 μM 2′,7′-dichlorodihydrofluorescein diacetate (DCFH; #C2938; Invitrogen, Rochester, NY, USA) and 10 μM 4-chloromethyl-6,8-difluoro-7-hydroxycoumarin ($CMF_2HC$; #C12881; Invitrogen) for 30 minutes, respectively. After washing in PBS-PVA three times, the fluorescence intensities were captured using a fluorescence microscope and analyzed with ImageJ software (NIH).

*6. MMP Assay*

Briefly, 4-cell-stage embryos at day 2 were washed three times with PBS-PVA and incubated in PBS-PVA containing 2 μM 5,5′,6,6′-tetrachloro-1,1′,3,3′-tetraethylbenzimidazolylcarbocyanine-iodide dye (JC-1; #M34152; Invitrogen) at 38.5 °C for 30 min. After washing with PBS-PVA three times, the red and green fluorescence intensities were captured using a fluorescence microscope. The average $\Delta\Psi m$ values of entire 4-cell-stage embryos were then calculated as the ratios of red fluorescence intensity to green fluorescence intensity using ImageJ software (NIH).

*7. Determination of ATP Levels*

The ATP levels in 4-cell-stage embryos were measured using an ATP Determination Kit (#A22066; Invitrogen) and a CentroPro LB 962 luminometer (Berthold Technologies, Bad Wildbad, Germany) according to the manufacturer's instructions with some modifications [20]. Before measurement, standard reaction solutions were prepared according to the manufacturer’s instructions and placed on ice in the dark. Briefly, 4-cell-stage embryos were collected in a 0.2 mL centrifuge tube containing 30 μL lysis buffer (20 mM Tris, 0.9% Nonidet-40, and 0.9% Tween 20) and lysed by ultrasonic shock. Then, 5 μL lysed sample solution was transferred into a 96-well plate and equilibrated for 10 sec. Subsequently, 200 μL standard reaction solution was added into the measurement well, and the light signal was integrated for 10 sec after a delay of 2 sec. The light intensity in the control group was arbitrarily set as 1, and the light intensity in the treatment group was then measured and expressed as values relative to the control group.

*8. Immunofluorescence*

Embryos at day 6 were washed with PBS, fixed for 30 min in 4% paraformaldehyde in PBS-PVA and permeabilized with 0.3% Triton X-100 in PBS-PVA for 30 min at room temperature. Then, the embryos were blocked for 1 h in 5% BSA in PBS-PVA at room temperature and incubated overnight at 4 °C with a primary antibody against

microtubule-associated protein 1 light chain 3 beta (LC3B; #ab63817; Abcam, Cambridge, MA, USA). Then, the embryos were washed three times in PBS-PVA, and incubated with a secondary antibody (#ab150073; Abcam) for 1 h at room temperature. The nuclei were stained with 10 µg/mL Hoechst 33342 for 10 minutes. Immunofluorescence was visualized and analyzed using a confocal laser scanning microscope (Carl Zeiss) and ImageJ software (NIH).

*9. qRT-PCR Analysis*

mRNA was extracted from 30 embryos on day 7 using the Dynabeads mRNA DIRECT Purification Kit (#61011; Invitrogen) according to the manufacturer's instructions without rRNA elimination. First-strand cDNA was synthesized using a TIANScript First Strand cDNA Synthesis Kit (#KR118; Tiangen Biotech Co., Beijing, China) following the manufacturer's instructions. For gene expression analysis, 10 µL of 2× SYBR Green RealMasterMix (#FP205; Tiangen) was used in a reaction mixture that comprised 1 µL (10 pmol) of each gene-specific primer, 1 µl of the cDNA sample, and 8 µL of deionized water in a final volume of 20 µL. Then, qRT-PCR was carried out under the following conditions: 300 s of polymerase activation at 95 °C followed by 40 cycles of denaturation at 95 °C for 10 s, annealing at 60 °C for 20 s, and elongation at 72 °C for 30 s. Gene expression was quantified using a Mastercycler ep Realplex system (Eppendorf, Hamburg, Germany) and the $2^{-\Delta\Delta Ct}$ method with 18S rRNA as the standard. All the primers used to amplify genes are listed in Supplementary Table S1.

*10. Western Blot Analysis*

On day 7, 30 blastocysts were placed in ice-cold RIPA Lysis buffer (#R0010; Solarbio, Beijing, China) containing protease inhibitors (#ST506; Beyotime) for total protein extraction heated at 95 °C for 10 min. Proteins were separated by SDS-PAGE and transferred to polyvinylidene fluoride membranes (0.45 µm; #IPVH00010; Millipore, Bedford, MA, USA). The membranes were transferred to 5% BSA blocking solution and placed on an oscillator for 2 h. The membranes were then placed in a chamber and incubated with a primary antibody at 4 °C overnight. After 3 washes, the membranes were incubated at room temperature for 2 h with secondary antibodies labeled with horseradish peroxidase. The antibodies information is shown in Supplementary Table S2. A Tanon 5200 (Tanon, Shanghai, China) system was used for imaging.
